# Comparison of pharyngeal airway volume in different skeletal facial patterns using cone beam computed tomography

**DOI:** 10.4317/jced.55033

**Published:** 2018-10-01

**Authors:** Abbas Shokri, Amirfarhang Miresmaeili, Ali Ahmadi, Payam Amini, Sepideh Falah-kooshki

**Affiliations:** 1 Dental Research Center, Department of Oral & Maxillofacial Radiology, Faculty of Dentistry, Hamadan University of Medical Sciences, Hamadan, Iran; 2 Dental Research Center, Department of Orthodontics, Faculty of Dentistry, Hamadan University of Medical Sciences, Hamadan, Iran; 3 Dentist, Department of Oral & Maxillofacial Radiology, Faculty of Dentistry, Hamadan University of Medical Sciences, Hamadan, Iran; 4 Department of Epidemiology and Reproductive Health, Reproductive Epidemiology Research Center, Royan Institute for Reproductive Biomedicine, ACECR, Tehran, Iran; 5 Department of Oral & Maxillofacial Radiology, Faculty of Dentistry, Kermanshah University of Medical Sciences, Kermanshah, Iran

## Abstract

**Background:**

This study aimed to compare the pharyngeal airway volume in class I,II and III skeletal malocclusion patients using cone beam computed tomography (CBCT).

**Material and Methods:**

This retrospective, cross sectional study was conducted on lateral cephalograms of 71 patients derived from their CBCT scans. Using the ANB angle, the patients were divided into class I,II and III malocclusion. Two observers used Dolphin 3D software to calculate the pharyngeal airway volume, airway area, minimum axial area, minimum area location, airway length and morphology. Data were analyzed using one-way ANOVA, Kruskal-Wallis test, Tukey’s test, Spearman’s correlation coefficient and multiple regression analysis.

**Results:**

The three skeletal classes were significantly different in airway volume, minimum axial area, mean airway area and airway morphology (*P*<0.05). Significant differences were found in airway volume and mean airway area between class II and III patients (*P*<0.05). The minimum axial area and airway morphology in class III patients were greater than those in class I and II patients (*P*<0.05). Every one unit increase in the ANB angle decreased the airway volume by 0.261 units. The effect of ANB angle on airway volume was statistically significant and it was shown that one unit increase in the angle decreased the airway volume by 453.509 units.

**Conclusions:**

A significant correlation exists between the skeletal facial pattern and upper airway dimensions. In our study, the total airway volume and the mean airway area of class III patients were larger than those in class II patients.

** Key words:**Cone-Beam computed tomography, malocclusion, pharyngeal airway volume.

## Introduction

The upper airway is a critical structure in the human respiratory system. The configuration and dimensions of the upper airway are dictated by the anatomical structures such as the soft tissue, muscles and the craniofacial skeleton surrounding the pharynx (1,2). The morphology of the pharynx affects the airway volume, facial growth pattern, risk of obstructive sleep apnea and masticatory pattern. Anatomical abnormalities of the soft tissue and craniofacial skeleton can change the pharyngeal airway volume (1). The pathological, physiological and morphological obstructive processes such as hypertrophy of the adenoids and tonsils, allergic and chronic rhinitis, stimulatory environmental factors, congenital nasal deformities, trauma to the nose, polyps and tumors are among the predisposing factors for the upper airway obstruction (2). In case of occurrence, a functional imbalance leads to mouth breathing, which can change the facial morphology and dental arch form, causing malocclusion (2).

Most previous studies in this respect had limitations since they evaluated the lateral cephalograms of patients. Lateral cephalometry provides a two-dimensional view of a three-dimensional (3D) structure and does not allow assessment of the volume of structures. Moreover, lateral cephalograms have other shortcomings such as distortion, low reproducibility due to problems in landmark identification, difference in magnification and superimposition of bilateral craniofacial structures (3). Techniques enabling accurate detection of changes in the upper airway consider the volume and morphology of the upper airway as the two main factors playing a role in normal growth and development of the craniofacial complex and correct treatment planning (4,5). Although computed tomography and cone beam computed tomography (CBCT) expose the patients to higher radiation dose compared to conventional digital radiography, CBCT is a highly acceptable imaging modality. CBCT has significantly lower patient radiation dose than computed tomography and has faster image acquisition (2,6,7).

Several studies have assessed the relationship of skeletal pattern and craniofacial morphology with the pharyngeal airway volume using CBCT and yielded controversial results. Some researchers believe that the respiratory pattern is an important etiologic factor responsible for change in craniofacial morphology while some others believe that change in facial morphology, as in the long face syndrome, has a genetic origin and the respiratory pattern is an enhancing factor (8,9). Evidence shows that type and severity of malocclusion can affect the size of the pharynx and increase the risk of obstructive respiratory diseases. Considering the significance of determining the morphology of the pharyngeal airway in different facial skeletal patterns and its effect on treatment planning, this study was carried out to evaluate the pharyngeal airway volume in different skeletal patterns in an Iranian population using CBCT. This study aimed to answer the question whether the skeletal pattern affects the airway volume.

## Material and Methods

This retrospective cross sectional study was conducted in Hamadan University of Medical Sciences, School of Dentistry in 2016-2017 and was approved by the ethics committee of this university (Res.Proj.16/35/1/5650/P). Sample size was calculated to be 71 patients. The exclusion criteria were patients under 18 years of age, those with edentulous areas, severe skeletal asymmetry, visible jaw fracture on CBCT scans, CBCT images taken with 6 and 9-inch fields of view, and also patients with CBCT scans on which, the upper half of the fourth cervical vertebra could not be seen. CBCT scans were taken using NewTom 3G Volume Scanner (QR srl, Verona, Italy) with the exposure settings of 110 kVp, 2.8 mA, 3.6 s time and 12-inch field of View. Images were processed using NNT Viewer software (Newtom, Verona, Italy). Using the Ray sum technique, lateral cephalograms were derived from the 3 dimention(3D) CBCT scans.

To determine the skeletal horizontal relationship of the jaws using NNT Viewer software, the ANB angle (the angle between the nasion, point A and point B) was determined. Accordingly, the patients were divided into three groups class I malocclusion (ANB angle between 1-5°), class II malocclusion (ANB angle>5°) and class III malocclusion (ANB angle<1°) (10).

There were 25 patients with class I, 24 with class II and 22 with class III malocclusion. Pharyngeal airway was divided into upper (nasopharynx) and lower (pharyngeal airway) segments.

**Figure 1:**
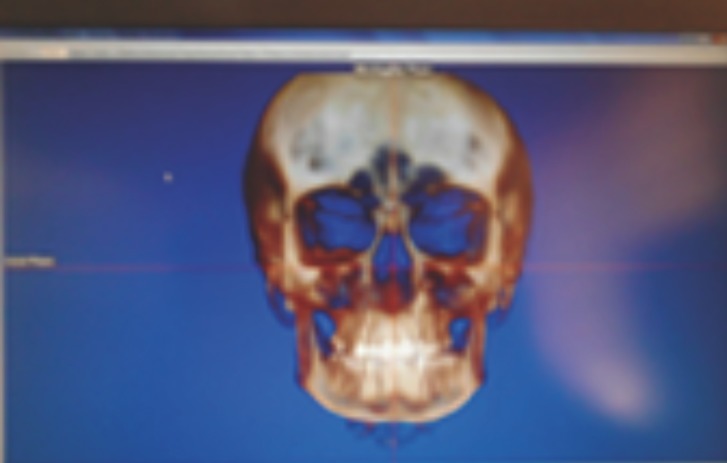
Calibration of the head in the frontal view.

Dolphin 3D software version 11.7 (Chatsworth, CA, USA) was used to analyze and calculate the volume of the pharyngeal airway. Dolphin software first standardized and calibrated the 3D head position in the axial, sagittal and frontal planes. In the frontal view, the mid-sagittal plane matched the skeletal midline and the coronal plane matched the line passing from the right and left inferior orbital rims (Fig. 1).

**Figure 2:**
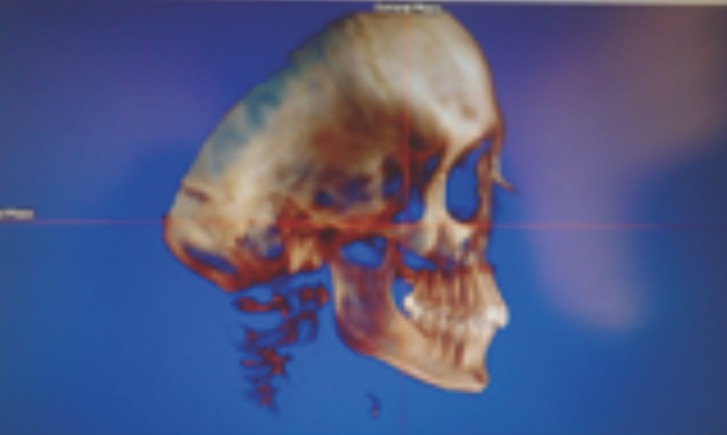
Calibration of the head in the lateral view.

On the lateral view, the Frankfurt plane was parallelized and matched the axial plane. Also, the coronal plane matched the line passing along the pterygomaxillary groove in the pterygopalatine fossa (Fig. 2). In asymmetric cases, calibration was done as close to the afore-mentioned reference planes as possible. Next, measurements and calculations of the airway volume were made using the sinus/airway feature of the Dolphin software.

-Analysis of the airway volume.

**Figure 3:**
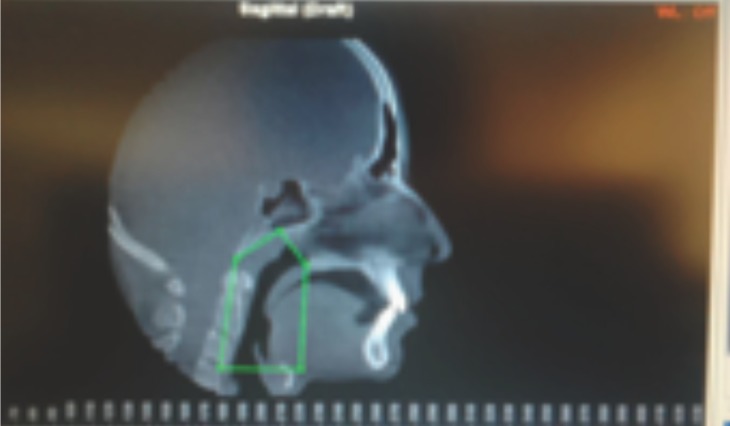
Outline of the airway .

To measure the airway volume, first we outlined the pharynx for calculation of airway volume by identification of the following landmarks:

- Hormion: The point of union of the sphenoid bone with the posterior border of the vomer (the most superior border of the pharyngeal airway) (Fig. 3)

- Posterior nasal spine (Fig. 3): The most inferior point of the skull base (basion)

- Line passing from the most superior and most anterior point of the fourth cervical vertebra and posterior wall of the pharynx parallel to the Frankfurt plane (Fig. 3).

After outlining the airway and parallelizing it with the Frankfurt plane, the slider tool of Dolphin 3D software was used to determine the sensitivity of the software for differentiation of the airway and the surrounding soft tissue and detecting the difference in resolutions of the pharyngeal airway. Since the size of the airway is not the same in different individuals, sensitivity is also variable. To confirm this hypothesis, we first determined airway sensitivity in 10 individuals and found that this hypothesis was correct. Thus, in order to minimize errors in outlining the airway, we initially determined the first sensitivity encompassing the entire airway area separately for each individual. In other words, in this sensitivity, the software was capable of showing the upper and lower compartments of the airway within the outlined area. This sensitivity was recorded as the minimum acceptable sensitivity for measurement of the airway volume in the respective individual. Next, using the same sensitivity, we changed the slices to manually add the areas not detected by the software as part of the airway by leaving seed points on the respective area. By doing so, we ensured that we did not miss any area of the pharyngeal airway in any slice (assessment of the airway space was done in coronal, axial and sagittal planes). After ensuring that the airway was correctly and completely outlined in all three planes, in minimum acceptable sensitivity, the airway volume was calculated using the software (it was done automatically by the software). The next airway sensitivities were determined similar to the minimum sensitivity, and airway volume was calculated in these sensitivities as well. The interval between the chosen sensitivities was 5 in order to have adequately high and differentiable resolution between the sensitivities. We measured the airway volume in different sensitivities as explained earlier until we gained a sensitivity value, which gave us a very large value for the airway value out of the confined border of the pharyngeal airway (for the soft tissue surrounding the pharynx). To find the final value for the airway volume, the two values closer to each other (smaller numerical difference) were chosen as the final values and the mean of these figures was calculated as the final pharyngeal airway volume for the respective individual.

**Figure 4:**
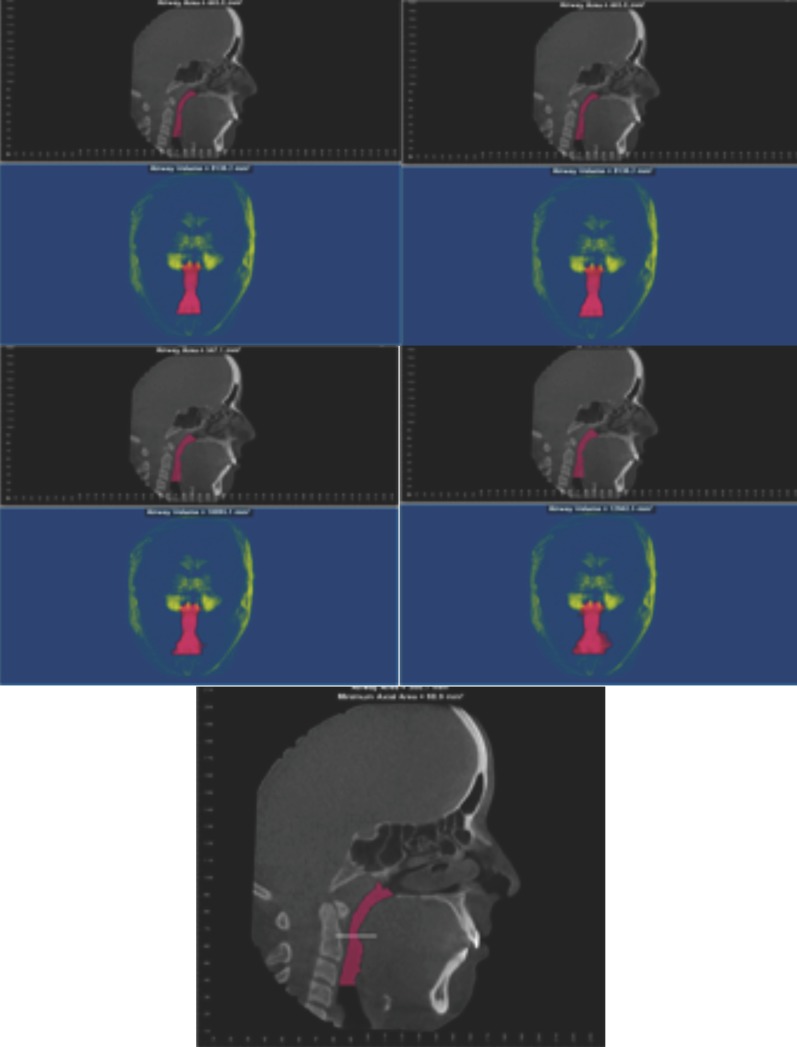
Calculation of airway volume in a patient; (A) minimum airway sensitivity (35), (B) airway sensitivity of 40.

In the next step, using the two chosen sensitivities, the airway area on the midline slice and the minimum axial area were automatically calculated by Dolphin software. Next, the mean of the values obtained in final sensitivities was determined as the final values for the airway area and minimum axial area. Figure 4 shows the range of different airway sensitivities for an individual.

After calculation of the volume, the mean airway area on a specific slice and the minimum axial area, the minimum area location of the lower pharyngeal segment was determined using the formula suggested by Holsbec (11): Location=upper airway length/total airway length.

The length of the upper airway was calculated as the distance between the upper border of the lower segment of the pharynx and the minimum area location of the axial section. In fact, airway location indicates minimum axial area (part of the airway with maximum narrowing) for evaluation in different skeletal patterns. To assess the morphology of the pharyngeal airway, first the mean airway area in different pharyngeal slices and then its morphology were calculated as follows (11).

Mean area: Volume/total airway length

Morphology= Minimum axial area/mean area 

The smaller the ratio, the more irregular and disperse the air distribution in the airway would be.

Two observers including a maxillofacial radiologist and an orthodontist evaluated the images in this study. The two observers determined the airway outline, anatomical landmarks and sensitivity for all lateral cephalograms. Two weeks later, all images were evaluated again by the two observers.

The mean and standard deviation values were reported for descriptive data, and diagrams and tables were drawn. The Kolmogorov-Smirnov test was used to analyze the normal distribution of data. One-way ANOVA was applied to compare continuous quantitative variables among the three skeletal patterns. This test evaluated the equality of variances for the mean continuous variables such as airway in the three skeletal patterns. Tukey’s test was applied for pairwise comparisons wherever the equality of variances was not met. The Spearman’s test was used to analyze the correlation of airway volume and minimum axial area. Multiple linear regression test was used to assess the effect of ANB angle, age and gender on airway volume. All statistical analyses were carried out using SPSS version 12 (SPSS Inc., IL, USA) with *P*<0.05 level of significance.

## Results

In this study, age, length of the upper airway and minimum axial location were not normally distributed but other variables showed normal distribution of data. ANOVA (parametric test) was used for the analysis of variables with normal distribution. The non-parametric Kruskal-Wallis test was used for age, length of the upper airway and minimum axial location ( Table 1).  Table 2 shows the frequency, mean and standard deviation of the airway variables.  Table 3 presents the frequency distribution and gender of skeletal class I, II and III patients.  Table 4 shows the frequency of sensitivity values used by Dolphin software to analyze the airway volume. Sensitivity in the range of 40-45 had the highest frequency. Also,  Table 4 shows that one sensitivity could not be used for calculation of airway volume in all individuals.

**Table 1:**
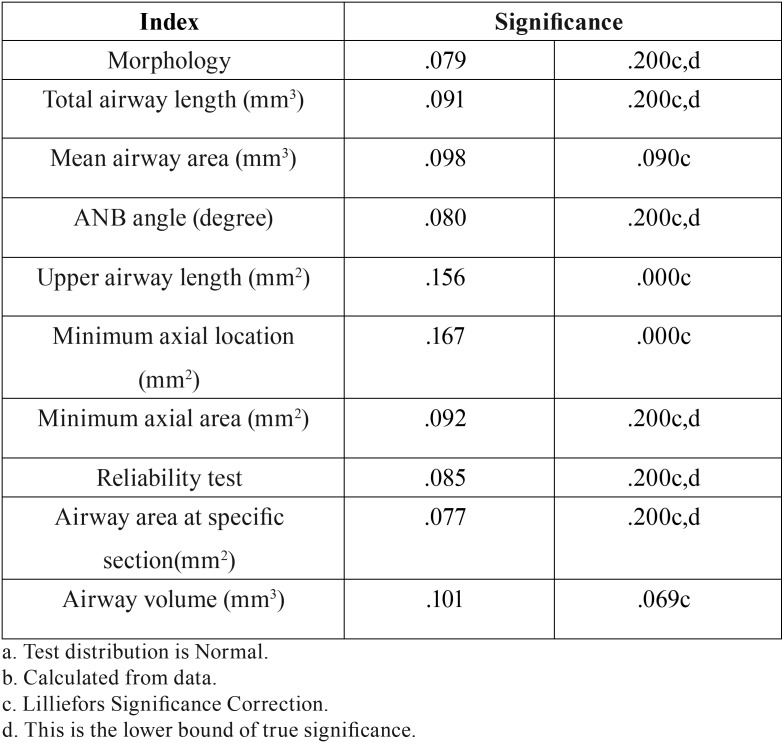
Evaluation of normal distribution of data using Kolmogorov-Smirnov test.

**Table 2:**
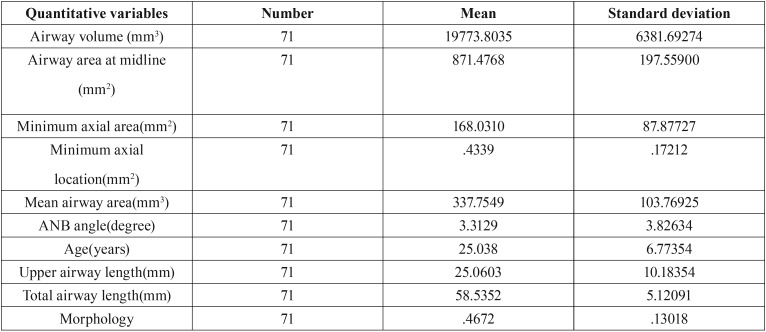
Frequency, mean and standard deviation of quantitative variables of the airway.

**Table 3:**
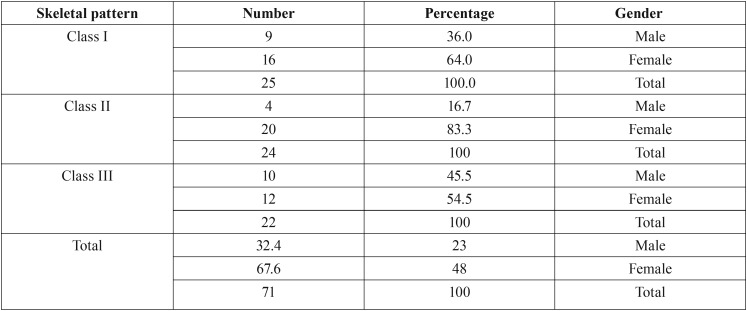
Frequency distribution of male and female patients with class I, II and III skeletal malocclusion.

**Table 4:**
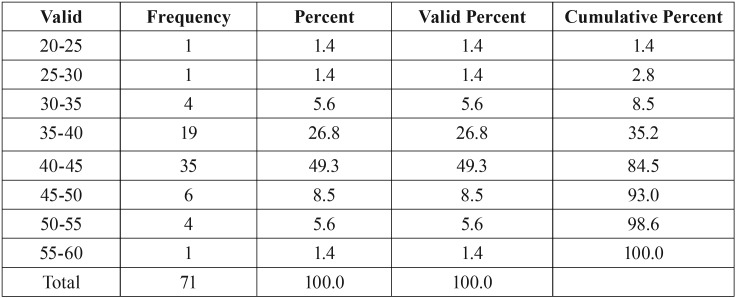
Frequency of sensitivity values used for airway volume analysis in Dolphin software.

One-way ANOVA was used to compare continuous quantitative variables namely airway volume, airway area on a particular slice (midline), minimum axial area, mean airway area, airway morphology and airway length in the three skeletal classes ( Table 5). ANOVA showed significant differences among the three skeletal classes in all the afore-mentioned variables except for the airway length ( Table 5).

**Table 5:**
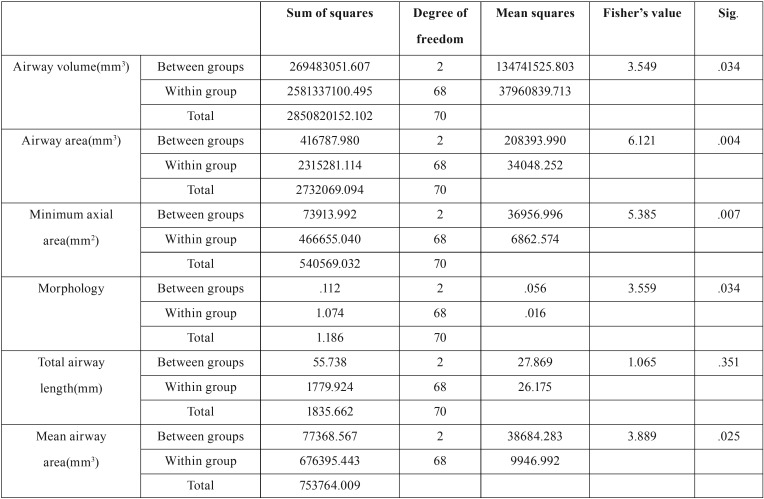
Between and within-group comparisons of quantitative variables of the airway using.

Tukey’s test was applied for pairwise comparisons of skeletal groups for airway volume, which showed a significant difference in this respect between skeletal class II and III patterns. Class III patients had greater airway volume than class I and II patients. Class I patients also had larger airway volume than class II patients. However, only the difference between class II and III patients was statistically significant in this respect. Class II patients had a smaller airway than class I patients but this difference was not significant ( Table 6). As shown in  Table 6, class II and III patients were significantly different in terms of airway area at the midline. This value in class II patients was smaller than that in class I patients, but not significantly. The minimum axial area in class III patients was significantly different from that in class II and I patients. The minimum axial area in class III patients was greater than that in class I and II patients. The difference in this respect between class I and II patients was not significant. Class II and III patients were significantly different in terms of the mean airway area, although the mean airway area of class III patients was greater than that in class I and II patients. The airway morphology of class III skeletal patients was greater than that of class I and II patients. The three skeletal groups were not significantly different in terms of total airway length.

**Table 6:**
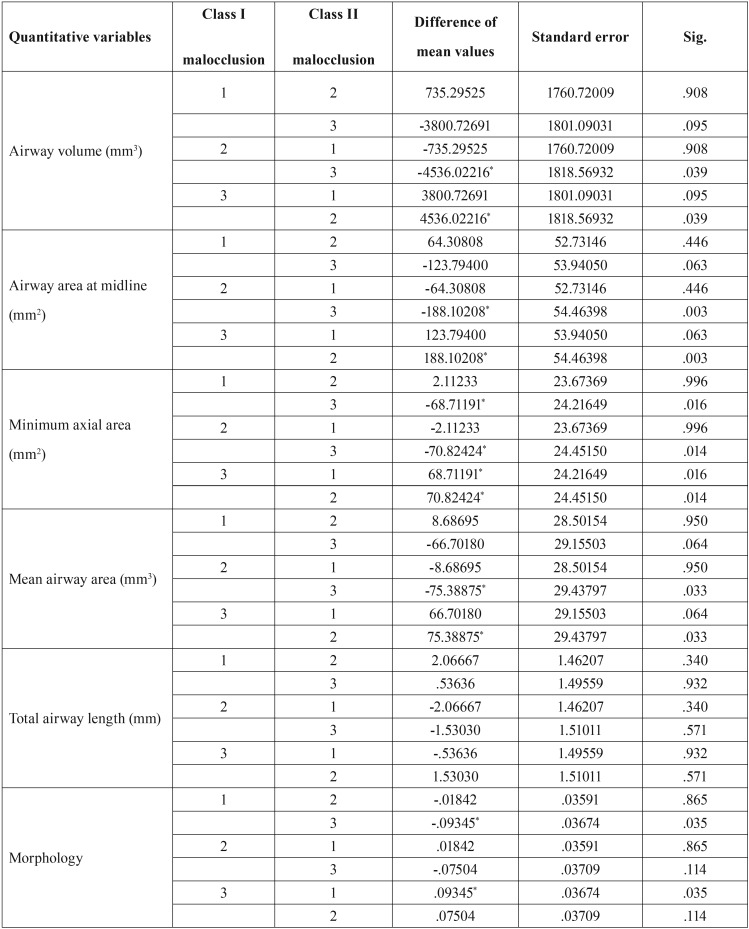
Pairwise comparison of skeletal patterns for quantitative variables using Tukey’s test.

The t-test was applied to compare the mean airway volume between males and females, which showed that although the airway volume was greater in males, this difference was not statistically significant.

The Pearson’s correlation coefficient showed that irrespective of the factors affecting the angle and volume of the airway, every one unit increase in the ANB angle decreased the airway volume by 0.261 units, which was statistically significant ( Table 7).

**Table 7:**
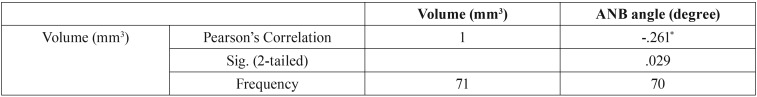
Correlation of ANB angle with airway volume.

The Kruskal-Wallis test showed differences in age, upper airway length and location among the three skeletal classes and revealed no significant difference ( Table 8).

**Table 8:**
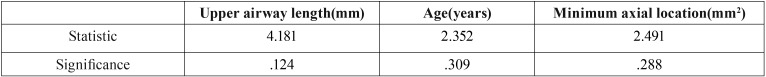
Comparison of upper airway length, age and minimum axial location.

Following unadjusted analysis of the effect of age, gender and angle on the airway volume, multiple linear regression was applied to assess the adjusted effect of each variable in presence of other variables on the airway volume ( Table 9). In absence of the variables in the model, airway volume was 21407.517; in the adjusted model, only the effect of angle on airway volume was found to be statistically significant and it was shown that one unit increase in the angle decreased the airway volume by 453.509 units ( Table 9). In this model, age and gender had no significant effect on airway volume. The final model was as follows:

**Table 9:**
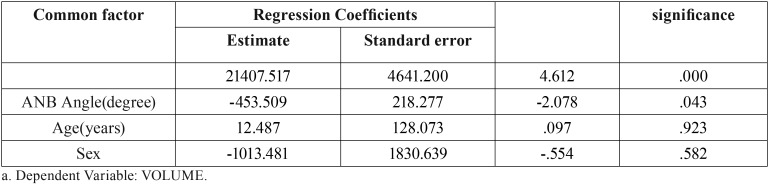
Modified effectiveness of age, sex and ANB angle on airway volume.

Volume=21407.517-453.509xangle

The intraclass correlation coefficient was applied to assess the reliability of repeated measurements, which was found to be 0.99.

## Discussion

This study compared the airway volume, mean surface area, minimum axial area, location and morphology among the three skeletal classes using Dolphin software. To compare the pharyngeal airway volume among the three skeletal classes, first, airway sensitivity was determined. The results of airway volume measurements according to the chosen sensitivity value are variable in different studies. Increased sensitivity value results in inclusion of the surrounding soft tissue in volume measurement (overestimation) while reduction in sensitivity value results in underestimation of airway volume and errors in calculation. The accuracy of airway measurements strongly depends on the chosen sensitivity value. It is noteworthy that the ideal sensitivity value for calculation of airway volume has not been standardized so far. In this study, in order to minimize errors, sensitivity was chosen separately for each individual. Evidence shows that type and severity of class II malocclusion affect the shape and size of the pharynx (12). Some researchers believe that a correlation exists between smaller airways and class II malocclusion (12). Also, it is believed that small airways can be the result of nasal obstruction (which is an anatomical incidence) and can compromise muscle function and change the facial growth pattern. According to this hypothesis, small airways are often associated with a small mandible. In this study, we found that skeletal class III patients had larger airways than other skeletal classes; although it only had a significant difference with skeletal class II pattern, and the difference between class I and II patients in terms of airway volume did not reach statistical significance.

The mean airway area in class III patients was larger than that in class II patients. The minimum axial area in class III patients was greater than that in class I and II patients but the difference in this respect between class I and II patients was not significant. Class III patients had more uniform airway distribution than other classes but no difference was found in the location of maximum airway narrowing (minimum axial area) among different skeletal patterns. Airway length was not significantly different either among the three groups. Our study showed a significant correlation between airway volume and maximum airway narrowing such that the larger the airway volume, the greater the minimum axial area would be. Researchers have divided class II malocclusions into different classes based on the size and position of the maxilla and mandible. Many researchers believe that skeletal class II patients have a much narrower airway than other classes. Given that a difference exists in shape and size of the airway among class I, II and III patients, it is important to find out what skeletal pattern would cause the greatest difference (13).

The results of previous studies on the airway volume are controversial. Claudino *et al.* (2) concluded that airway volume in class II patients is smaller than that in class I and III patients, although they did not mention the mean value of volumes they calculated. Fagala (14) evaluated class I and II patients and found no significant difference in airway volume between the two groups, which was similar to our findings. This finding was in contrast to that of Claudino *et al.*, which may be due to different methodologies. In the study by Claudino *et al.*, (2) the sensitivity value was fixed at 25 while Fagala (14) chose the sensitivity value of 45. The age range was close in the two studies; however, they obtained different results, which may be due to different sensitivity values (at least partly). One major drawback of the studies by Fagala and Claudio *et al.*, was that they used a fixed sensitivity value for all patients. Fagala refuted the hypothesis regarding the presence of a correlation between skeletal malocclusion and airway volume. However, he did not evaluate class III patients in his study. One major advantage of the study by Fagala over our study was large sample size (n=160) (14). He divided the lower airway into three segments using PNS and soft palate planes, epiglottis and the third cervical vertebra in order to assess the pharyngeal airway separately in each segment. However, it should be noted that dividing the airway into different segments and their comparison may cause errors in measurement since the location of landmarks may vary in different individuals. Kula *et al.* (15) compared airway volume among the three skeletal classes and found no significant difference. In their study, similar to ours, the lower border of the airway was the most superior point of fourth cervical vertebra. They even considered the nasal cavity for measurement of the airway volume. However, the mean airway volume measured in their study was smaller than the airway volume calculated in our study. This difference was probably due to the method of airway measurement. It seems that the method of selection of sensitivity value was the main reason for this difference. Dadbin *et al.* (16) performed airway analysis using Dolphin software and reported larger airway volume in class III patients. Also, they showed that class II patients had smaller airway volume than others. The mean airway volume reported in their study was much larger than the value reported in our study, which was probably due to the selection of large sensitivity values for measurement of airway volume. Grauer *et al.*, and Kim *et al.* (17,18) showed that class II patients had smaller airway volume than other skeletal patterns. However, such small airway volume in the study by Kim *et al.*, (18) seems to be due to statistical error as the result of small sample size. A study population comprising of 27 patients does not have adequately high statistical power to correctly confirm the presence of a difference between two groups. In contrast to studies by Grauer *et al.*., and Kim *et al.*, Alves *et al.* (17-19) reported that the airway volume was the same in class II and III patients and concluded that most airway dimensions are not affected by the type of malocclusion. However, similar to the studies by Grauer *et al.*, and Kim *et al.*, Alves *et al.* had a small sample size, which questions the accuracy of their results. They did not mention how they determined the sensitivity value either.

Several studies have evaluated the mean airway area and minimum axial area. Some studies on craniofacial morphology and pharyngeal airway stated that the risk of airway collapse was high in skeletal class II patients. Claudino *et al.* believed that class II patients had higher risk of developing obstructive sleep apnea compared to patients with other skeletal patterns. However, we did not find evidence to support this hypothesis in our study because the minimum axial area to increase the risk of airway collapse is under 50 mm2, and such low values were only found in two class I and two class II patients in our study. Moreover, the mean value of minimum axial area in these two skeletal patterns confirmed that the obtained values were much higher than this range. In a study on obstructive sleep apnea, the mean value of minimum axial area was around 146.9 mm2 (20). This confirms our findings since the values obtained in class I and II patients in our study were close to this value. The situation was even better for class III patients, and small minimum axial area value was not found in any class III patients. These patients had a higher mean value of minimum axial area than patients with class I and II skeletal patterns. In our study, no significant difference was noted between class I and II patients in terms of the minimum airway area. Kula *et al.*, in contrast to our study and that of Claudino *et al.*, found no difference in the minimum axial area among different skeletal classes (2,15). But, Alves *et al.*, in contrast to Kula *et al.* found a significant difference in the minimum axial area between class I and II patients, which was not in line with our findings (19). Their study was different from ours in that they did not include the nasopharynx and also used epiglottis soft tissue plane as the most inferior border of airway. The hard palate plane was used as the superior border of airway. These differences may explain the difference between our results and theirs; but most importantly, Alves *et al.* did not mention how they calculated the sensitivity value.

The upper airway morphology is an important parameter predicting the risk of airway obstruction. The smaller this ratio, the more irregular and disperse the pattern of airflow distribution in the pharynx would be. In our study, a larger value was obtained for class III patients compared to others, which indicates better airway distribution and lower risk of airway obstruction compared to other patterns. This variable was only calculated in our study and that of Claudio *et al.* They found no difference in airway morphology of patients with different skeletal patterns. However, they concluded that in the hypopharynx area, airway morphology of class II patients was less frequent than that of other patterns (2).

Minimum axial location indicates the location of minimum axial surface area of the airway. In our study, the three skeletal classes were not significantly different in this respect; although the minimum axial location in most of our patients was in the oropharynx. In the study by Claudino *et al.*, (2) the minimum axial location in class II patients was in the oropharynx and they believed that this area had the highest risk of obstruction. However, in our study, minimum axial location had higher variability. Kula *et al.*, (15) similar to our study, found no significant difference in this respect among different skeletal classes.

Controversial results have also been reported regarding the ANB angle. Alves *et al.* (21) showed that class I patients (with ANB angle between 2-5°) had larger airway dimensions than class II (ANB angle >5°) patients and concluded that ANB angle affects the airway volume. Claudino *et al.* (2) stated that a significant correlation exists between the airway volume and ANB angle. But Kula *et al.*, and Alves *et al.* (15,21) found no significant association between airway volume and the ANB angle. Our study results confirmed the findings of Claudio et al., and showed that each one degree increase in size of the ANB angle would decrease the airway volume by 453 mm3.

We also evaluated the effect of age on airway volume. According to Schendel *et al.*, (5) airway dimensions increase until the age of 20 and remain constant thereafter. Fagala (14) concluded that the airway volume increases with aging; this finding seems logical since their study was conducted on 8-14 year olds (within the developmental age range). In our study, the mean age of patients was 25 years (range 18 to 50 years) and age had no significant effect on airway volume. Other studies on airway volume in males and females reported different results. Grauer *et al.* (22) believed that class III males had a much larger nasopharynx than females. Alves*et al.* (21) found a significant difference in airway volume of males and females. But Handelman and Osborne (23), Klein *et al.*, (24) and Solow *et al.*, (25) showed that gender had no significant effect on airway dimensions. In our study, airway volume was not significantly different between males and females. However, airway volume in males was larger than that in females. Our sample size was limited in this study and number of males was half the number of females. This may explain the lack of significant difference in this regard in our study.

In our study, it was hypothesized that a difference exists in dimensions of the pharyngeal airway of class II malocclusion patients compared to other skeletal classes. It was hypothesized that class II patients would have smaller airways due to smaller size of the mandible and less space for the pharynx. However, some studies rejected this hypothesis and found no correlation between skeletal pattern and airway volume. But our study found a significant correlation between the skeletal pattern and airway dimensions. In our study, class II and I patients were not significantly different in terms of airway volume but by an increase in ANB angle, the pharyngeal airway volume significantly decreased. If we had a larger sample size or had chosen class II samples with larger ANB angles, we might have obtained a significant difference in class I patients as well.

Despite the clinical significance of obstructive sleep apnea, generalization of these results to obstructive sleep apnea patients is not possible because in our study, CBCT images were obtained of patients while awake and there is no way to find out whether their tongue was in the standard position although patients were radiographed in supine position. This is an advantage compared to patients radiographed in standing position. In studies that radiographed patients in standing position, airway volume was overestimated compared to studies on patients radiographed in supine position.

A significant correlation exists between different skeletal patterns and upper airway dimensions. Total airway volume (sum of nasopharynx, oropharynx and hypopharynx) and the mean airway area of class III patients were larger than those in class II patients. In class I and II skeletal patterns, the difference in airway volume was not significant. The minimum axial area in class III patients was greater than that in class I and II patients. Airway and airflow distribution along the pharynx in class III patients was more uniform than that in class I and II patients. Also, the minimum axial area in different skeletal patterns was not significantly different. This indicates that the narrowest part of the pharyngeal airway is not always located in the same region. The ANB angle is an important factor affecting airway dimensions. Further studies on airway volume, in association with sleep studies, are required to radiograph patients while asleep. Combining this information with nasopharyngeal dimensions and BMI can help in better understanding of sleep apnea.
